# Global Outbreaks and Origins of a Chikungunya Virus Variant Carrying Mutations Which May Increase Fitness for *Aedes aegypti*: Revelations from the 2016 Mandera, Kenya Outbreak

**DOI:** 10.4269/ajtmh.18-0980

**Published:** 2019-03-11

**Authors:** Irina Maljkovic Berry, Fredrick Eyase, Simon Pollett, Samson Limbaso Konongoi, Michael Gordon Joyce, Katherine Figueroa, Victor Ofula, Helen Koka, Edith Koskei, Albert Nyunja, James D. Mancuso, Richard G. Jarman, Rosemary Sang

**Affiliations:** 1 Viral Diseases Branch, Walter Reed Army Institute of Research, Silver Spring, Maryland;; 2 United States Army Medical Research Directorate – Kenya, Nairobi, Kenya;; 3 Center for Virus Research, Kenya Medical Research Institute, Nairobi, Kenya;; 4 Military HIV Research Program, Walter Reed Army Institute of Research, Silver Spring, Maryland;; 5 Henry M. Jackson Foundation for the Advancement of Military Medicine, Inc., Bethesda, Maryland

## Abstract

In 2016, a chikungunya virus (CHIKV) outbreak was reported in Mandera, Kenya. This was the first major CHIKV outbreak in the country since the global reemergence of this virus in Kenya in 2004. We collected samples and sequenced viral genomes from this outbreak. All Kenyan genomes contained two mutations, E1:K211E and E2:V264A, recently reported to have an association with increased infectivity, dissemination, and transmission in the *Aedes aegypti* vector. Phylogeographic inference of temporal and spatial virus relationships showed that this variant emerged within the East, Central, and South African lineage between 2005 and 2008, most probably in India. It was also in India where the first large outbreak caused by this virus appeared, in New Delhi, 2010. More importantly, our results also showed that this variant is no longer contained to India. We found it present in several major outbreaks, including the 2016 outbreaks in Pakistan and Kenya, and the 2017 outbreak in Bangladesh. Thus, this variant may have a capability of driving large CHIKV outbreaks in different regions of the world. Our results point to the importance of continued genomic-based surveillance and prompt urgent vector competence studies to assess the level of vector susceptibility and virus transmission, and the impact this might have on this variant’s epidemic potential and global spread.

## INTRODUCTION

In May 2016, the Kenyan Ministry of Health (KMoH) reported an outbreak of chikungunya virus (CHIKV) in Mandera County on the border with Somalia. During this time in Somalia, outbreaks of CHIKV were occurring in the neighboring Bula Hawa region, originating from Mogadishu. In Mandera town, 1,792 cases were detected, and an estimated 50% of the health work force was affected by this virus. A cross-border joint response was coordinated between Kenya and Somalia to control the outbreak.^1^ This was the first reported outbreak of CHIKV in Kenya since 2004.

The previous large CHIKV outbreak in Kenya occurred in Lamu Island in 2004, with an estimated 75% of the population infected.^2^ The disease also spread to the coastal city of Mombasa by the end of 2004, and further to the Comoros and La Réunion islands, causing large outbreaks in 2005–2006. On La Réunion island, unusual clinical complications were reported in association with CHIKV infection, and viral isolate sequences revealed the presence of an alanine-to-valine mutation in the E1 glycoprotein at position 226 (E1:A226V).^3^ This specific mutation was shown to confer significant increase in CHIKV infectivity for the *Aedes albopictus* vector, which was also the dominant mosquito species suspected to be responsible for the transmission of CHIKV on the island of La Réunion.^4,5^ Since then, the E1:A226V mutation has been observed in many of the genomes in the CHIKV lineage, spreading in the East, Central, and South African region (ECSA lineage), and has been shown to have emerged through convergent evolution in at least four different occasions.^6^

The remarkable emergence and spread of CHIKV adaptation to the *Ae. albopictus* vector prompted additional studies on the genetic plasticity of this virus, looking for additional biomarkers associated with virus transmission capacity, fitness, and pathogenicity.^6–8^ Mutations with the ability to enhance infection in this vector are of increased importance, as *Ae. albopictus* is rapidly expanding throughout the world.^9^ The *Ae. albopictus* mosquito is believed to have originated in Asia and is today most commonly found in east Asia. *Aedes albopictus* is also common in some parts of South and Southeast Asia, India, and Africa, and it has shown increased spread to regions with lower temperatures, such as southern Europe, southern Brazil, northern China, and the northern United States.^9^ In Europe, this vector has been associated with autochthonous transmission of CHIKV.^10^

Although some of the more recent CHIKV outbreaks have been transmitted by *Ae. albopictus*, most of the CHIKV transmissions in the world are associated with *Aedes aegypti*. *Aedes aegypti* is a container-breeding, domesticated mosquito mainly found in urban areas and feeding largely on humans during early and late daytime hours. *Aedes aegypti* originated from the ancestral zoophilic *Ae. aegypti formosus* native to Africa. *Aedes aegypti* now is most common in tropical and subtropical regions, such as Africa, India, Southeast Asia, and Australia.^9^ Recently, two mutations, E1:K211E and E2:V264A, have been reported in the CHIKV to be associated with increased fitness to *Ae. aegypti* vectors.^11^ These two mutations, in the background of the wild-type E1:226A, are believed to increase virus infectivity, dissemination, and transmission in *Ae. aegypti*, with no impact on virus fitness for the *Ae. albopictus* vector.^6,11^ E1:K211E was first observed in genomes sampled in 2006 from the Kerala and Puducherry regions, India, and simultaneous presence of both mutations was first observed in 2009–2010 in Tamil Nadu and Andhra Pradesh, India.^12,13^

In Kenya, the predominant CHIKV vector is *Ae. aegypti*, and vector competence studies using local vector populations have shown it is capable of transmitting the virus in this region.^14^ Given the recent large outbreak of CHIKV in the rural setting of Mandera County, Kenya, we analyzed CHIKV genomes sequenced from this outbreak for the presence of adaptive mutations associated with both *Ae. albopictus* and *Ae. aegypti*. Along with estimating origins and the time of emergence of a variant that carried two of these previously reported mutations, we also estimated time and origins of the Mandera CHIKV outbreak. Our results indicate the spread of a CHIKV that carries mutations previously associated with increased fitness for *Ae. aegypti* in Kenya. We also show that this variant is now connected with new large outbreaks in other regions of the world.

## MATERIALS AND METHODS

### Ethics statement.

The study was carried out on a protocol approved by the Walter Reed Army Institute of Research (#2189) and Kenya Medical Research Institute’s (KEMRI) Scientific and Ethics Review Unit (#3035) as an overarching protocol guiding investigation and reporting of arbovirus/hemorrhagic fever outbreaks in Kenya. Because the blood samples were collected from an outbreak and no human data were collected, this was deemed to be a nonhuman research study and no consent was required. The protocol was approved for additional analysis of outbreak samples and for publication of results.

### Samples and sequencing.

From May 2016, following reports of widespread incidence of febrile illness with severe joint pains in Mandera (a city at the border with Somalia) and its environs, samples were collected from suspected cases of all ages and gender, using standard practices by the KMoH staff. The case definition used was “any patient presenting with sudden onset of fever > 38.5°C, with severe joint/muscle pains and headache within the last 3–5 days within Mandera County, the person should either be a resident of or visiting Mandera.” Chikungunya infection was confirmed by CHIKV-specific reverse transcription polymerase chain reaction (RT-PCR) and partial genome Sanger sequencing at the KEMRI laboratories. Vero cell culture inoculations were performed on the samples to obtain isolates for in-depth studies. For method details on infection confirmation, virus isolation, and Sanger sequencing, see Supplemental Appendix 1. A subset of chikungunya-confirmed positive samples from acutely ill patients were further subjected to high throughput full-genome sequencing at the KEMRI laboratories. The prepared complementary DNA (cDNA) was quantified using Qubit 3.0 fluorimeter and the dsDNA HS Assay Kit (Life Technologies–Thermo Fisher Scientific, Saint Aubin, France). The cDNA was fragmented enzymatically and tagged using the Nextera XT DNA Library kit (Illumina, San Diego, CA). Each sample was assigned a unique barcode sequence using the sequence libraries prepared with the Nextera-XT kit (Illumina), following the protocols and reagents supplied by Illumina, and sequenced on the MiSeq platform according to the manufacturer’s instructions (Illumina). Ten full genomes and five partial genomes were assembled by combining both de novo and reference mapping assemblies. De novo assemblies were performed using Abyss and Trinity, and reference mapping was performed using ngs_mapper.^15–17^ Sequences have been submitted to the GenBank under accession numbers MH423797–MH423811.

### Phylogenetic analyses.

All full-genome CHIKV genomes were downloaded from the GenBank and curated in TempEst.^18^ The curation consisted of inference of a neighbor joining tree followed by linear regression of root to tip distances, given the sampling time. Genomes with too much or too little divergence as would be expected, given their root to tip distance and collection date, were considered as outliers and were removed from the dataset. In addition, all genomes with long stretches of Ns, genomes without collection date, and genomes without sampling location were also removed from the reference dataset. To determine the CHIKV lineage of the assembled genomes from Mandera, Kenya, these sequences were aligned to the constructed reference dataset (*n* = 466), using MEGAv7.^19^ This large dataset was also scanned for the presence of recombination using Recombination Detection Program version 4, with a minimum of three methods with significant (*P* < 0.05) recombination signal required to call a genome recombinant.^20^ Maximum likelihood (ML) trees were inferred using PhyML and RaxML, with general time reversible + gamma + invariant sites (GTR+G+I) (GTR+G for RaxML) model of evolution, as determined by jModelTest2.^21–23^ Node confidence values were derived by approximate likelihood-ratio test (aLRT) (PhyML) and bootstrap of 500 (RaxML). After evaluating the temporal structure using TempEst, the trees were used for an informed down-sampling of the dataset. For this analysis, only ECSA lineage genomes were kept as reference, identical genomes from the same location and sampling year were removed, as were genomes from a phylogenetically distant sub-clade only found to spread in Asia. The down-sampled dataset (ECSA lineage only, *n* = 115) was analyzed using the BEAST package v1.8.3, with GTR+G+I model of substitution, bayesian skyline plot, relaxed lognormal molecular clock, and asymmetrical trait (location) distribution.^24^ BEAST was run for 800 million generations, with subsampling every 80,000 and 10% burn-in. The maximum clade credibility (MCC) tree was summarized using TreeAnnotator. Ancestral states were reconstructed using ML, and all amino acid mutations at each node for the trees were mapped using TreeTime.^25^ Because viral genomes from several additional recent CHIKV outbreaks form India and Bangladesh were only sequenced in their E1 gene, we also inferred the ML tree of the partial E1 region including these sequences, using PhyML and TN93+G+I model of evolution, as determined by jModelTest2.

### Selection and protein structure analyses.

Selection analyses were performed using the HyPhy package.^26^ The presence of site-specific selection was determined by a likelihood approach using FEL and by Bayesian method FUBAR with a probability level threshold of 0.95.^27,28^ Episodic selection was investigated using MEME, and any selection acting on tree branches was determined by aBSREL.^29,30^ Selection analyses were performed on the large full-genome dataset used for the ML trees (ECSA only) and the small dataset used for BEAST analyses. FUBAR was performed on separated structural and nonstructural genes from both datasets because of their large sizes. Positive selection on a site was determined present if the site was found to be under selection by all three methods that determine site-specific selective pressures (FEL, FUBAR, and MEME). Structure figures of CHIKV E1E2 (Protein Data Bank ID:3J2W^31^) were visualized and prepared using PyMOL (The PyMOL Molecular Graphics System; DeLano Scientific, Palo Alto, CA).

## RESULTS

### Chikungunya virus causing the 2016 outbreak in Mandera, Kenya, was introduced in 2015.

**Figure 1.**
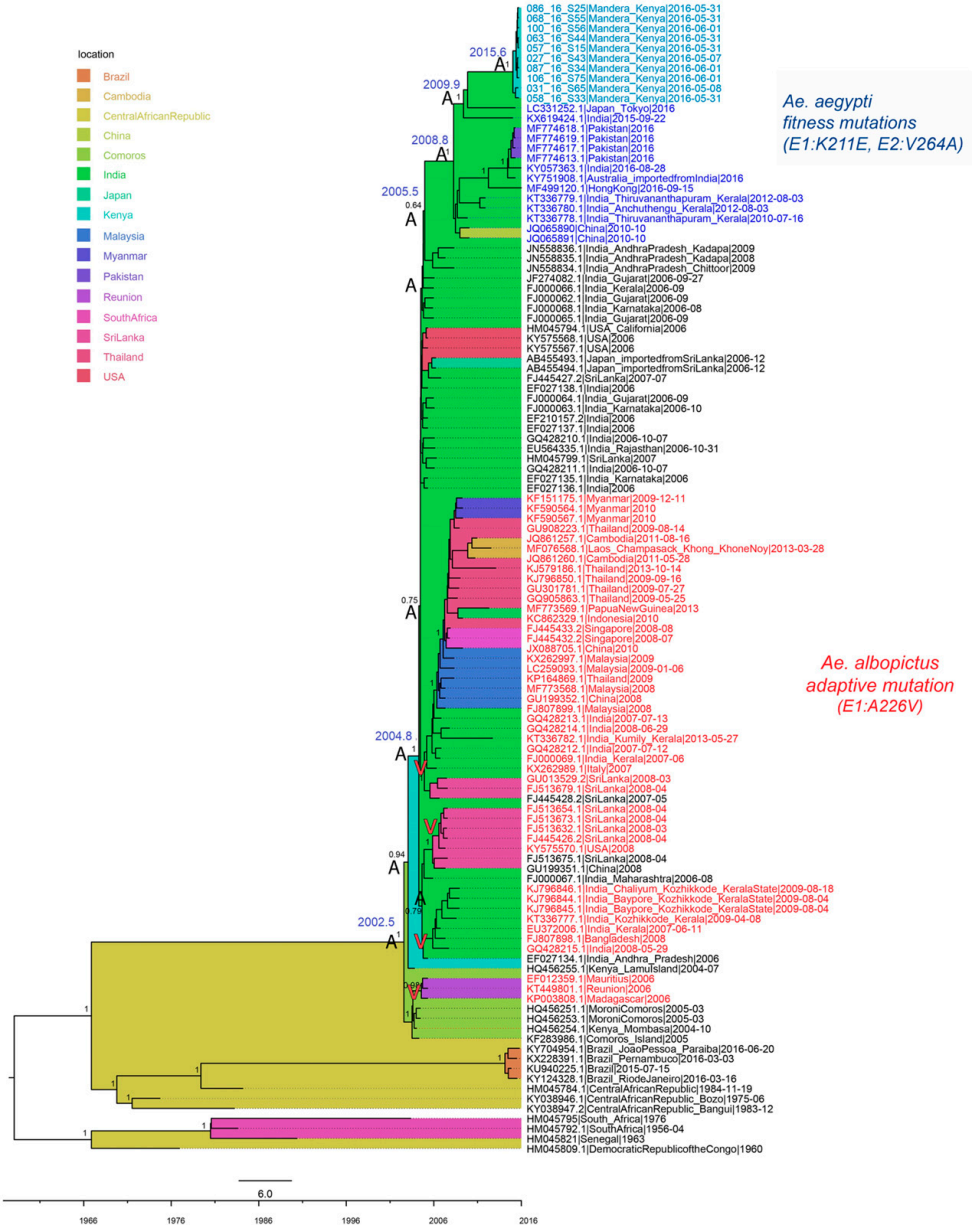
Full-genome maximum clade credibility tree of the chikungunya virus East, Central, and South African lineage. Estimated location origin is marked in colored tree background, according to the legend. Taxa in red text represent genomes containing the *Aedes albopictus*–adaptive E1:A226V mutation, whereas all other taxa contain the wild type. Taxa in blue (light blue for Kenya) represent genomes with the E1:K211E and E2:V264A mutations, previously associated with increased fitness in *Aedes aegypti*. Most important node supports are shown, as well as the estimated times of the most recent common ancestors (TMRCAs) for nodes of interest. Ancestral amino acid states (A and V) at position E1:226 are plotted on the nodes to illustrate evolutionary paths of the *Aedes albopictus* and *Ae. aegypti* mutation variants. This figure appears in color at www.ajtmh.org.

A total of 15 samples from the CHIKV outbreak in Mandera, Kenya, collected in May and June 2016, were sequenced. Ten of the sequenced samples produced full CHIKV genomes and five of the samples had partial genomes. No recombination was detected in the genomes. Maximum likelihood trees inferred by PhyML and RaxML were concordant and showed that all Kenyan genomes belonged to the ECSA lineage of CHIKV. Consistently with their outbreak origins, the genomes from Mandera clustered in a monophyletic clade defined by very short branches, indicating limited genetic diversity (Figure 1). The Kenyan cluster was most closely related to genomes from Japan and India; however, the long branch leading to this cluster indicated a probable lack of sampling of viruses more related to the Kenyan outbreak. Two genomes from the CHIKV outbreak in Kenya in 2004 were located more basally in the tree and did not cluster together with the viruses causing the Kenyan outbreak of 2016. The most recent common ancestor (MRCA) of the Kenyan 2016 viruses existed in mid-2015 (2015.6; highest posterior density (HPD) 95% = 2015.1–2015.9), making this the latest possible time point of introduction of this virus into Mandera, Kenya. The ancestor shared with the most closely related genomes from Japan and India existed in late 2009 (2009.9; 95% HPD = 2008.6–2011.5) and was estimated to have originated in India. These results indicate that the CHIKV causing the 2016 outbreak in Kenya was introduced into this region by 2015. Although the results suggest that the Mandera virus originated from a virus that existed in India in 2009, the long branch leading to the Kenyan cluster indicates missing data. Thus, India might not have been the direct source of the CHIKV introduction into Kenya, and more sampling is needed to, with greater precision, determine the exact origin of this introduction.

### Mutations associated with possible increased fitness to *Ae. aegypti* emerged by 2008.

Careful analyses of amino acid mutations associated with vector adaptation revealed that all Kenyan viruses contained two mutations, E1:K211E and E2:V264A, as well as the E1:226A background amino acid (Table 1). In the background of E1:226A, E1:K211E and E2:V264A have recently been correlated with enhancement of CHIKV fitness for the *Ae. aegypti* vectors from India. Further investigation of amino acids from the genomes surrounding the Kenyan samples in both the ML and MCC phylogenetic trees revealed a cluster of genomes, sampled from various regions of the world (Asia, Africa, and Australia), also containing the two *Ae. aegypti* fitness-associated mutations in the background of E1:226A (Figure 1, Table 1). These two amino acid changes were the only ones that characterized this cluster.

**Table 1:** Changes in positions previously associated with vector competence and pathology of chikungunya virus

Protein	Amino acid substitution	Phenotype	Asian lineage	ECSA lineage without *Aedes aegypti*–adapted cluster	ECSA *Ae. aegypti*–adapted cluster	Kenya
E1	A98**T**^6,7^	Epistatic covariant on E1:A226V	**T**	A	A	A
E1	A226**V**^4^	Enhanced infection of *Aedes albopictus*	A	A, **V**	A	A
E1	K211**E**^11^	Enhanced fitness in *Ae. aegypti* in background of E1:226A	**E**	K, T, N	**E**	**E**
E2	V264**A**^11^		V	V	**A**	**A**
E2	R198**Q**^6^	Enhanced infection of *Ae. albopictus*, synergistic with E3:18F in background of E1:226V	R	R, **Q**	R	R
E2	L210**Q**^6,8^	Enhanced infection of *Ae. albopictus,* secondary to E2:A226V	L	L, **Q**	L	L
E2	K233**E/Q**^6^	Enhanced infection of *Ae. albopictus*	K	K, **E**	K	K
E2	K234**E**^6^	Enhanced infection of *Ae. albopictus*	K	K	K	K
E2	K252**Q**^6^	Enhanced infection of *Ae. albopictus*—secondary mutation	K	K, **Q**	K	K
E3	S18**F**^6^	Enhanced infection of *Ae. albopictus*, synergistic with E2:R198Q	S, **F**	S, **F**	S	S
nsP1	G230**R**^32^	Increase replication in *Ae. albopictus* in combination with nsP3:524*	G, **R**	G, **R**	G	G
	V326**M**^32^		V, **M**	V	V	V
nsP3	*524**R**^33^	Attenuation of arthritis and pathology	*, L, C, **R**	*, C, **R**	*	*

ECSA = East, Central, and South African. Amino acids associated with each phenotype are shown in bold underlined font. Only available full genomes from each lineage were compared. Amino acids associated with each phenotype are highlighted in the table in bold and underscored font.

* Opal STOP codon.

The cluster containing the genomes with E1:K211E and E2:V264A mutations consisted of viruses from two different outbreaks, Kenya in 2016 and Pakistan in 2016, and additional genomes from India sampled between 2010 and 2016, and Japan, Hong Kong, and Australia, all sampled in 2016. The MRCA of this cluster was estimated to have existed in India since late 2008 (2008.8; 95% HPD = 2007.9–2009.5), indicating that the virus with dual E1:K211E and E2:V264A mutations emerged by the end of 2008 in this area of the world. The cluster shared a common ancestor with genomes from India, which did not contain the two suggested *Ae. aegypti* fitness mutations, in mid-2005 (2005.5; 95% HPD = 2005.0–2006.1). Although the node support for the ancestor of viruses with and without the two mutations was low, probably because of lack of sampling that would reveal the exact evolutionary relationships, it was estimated to have existed in India because of well-supported ancestral nodes preceding this one. Thus, these results indicated that the variant with possible increased fitness to *Ae. aegypti* most probably arose in India sometime between 2005 and 2008. The background E1:226A amino acid (non-red taxa, Figure 1) predominated in this part of the tree, whereas the *Ae. albopictus*–adaptive E1:226V amino acid (red taxa, Figure 1) was mainly found in the sub-lineage containing genomes from Southeast Asia. Genomes from India sampled in years 2007–2009 were found containing E1:226A and E1:226V mutations, meaning that this country experienced simultaneous spread of both the ancestral wild-type strain and the *Ae. albopictus*–adapted strain. None of the *Ae. albopictus*–adapted viruses contained the E1:K211E and E2:V264A changes, which were found occurring only in the presence of the E1:226A wild-type residue. Ancestral state reconstruction further revealed that the variant with the dual E1:K211E and E2:V264A *Ae. aegypti* fitness mutations did not arise from the *Ae. albopictus*–adapted strain (Figure 1). Rather, the *Ae. aegypti* variant evolved directly from the E1:226A wild type that was circulating in India at the same time as the E1:226V *Ae. albopictus*–adapted virus.

**Figure 2.**
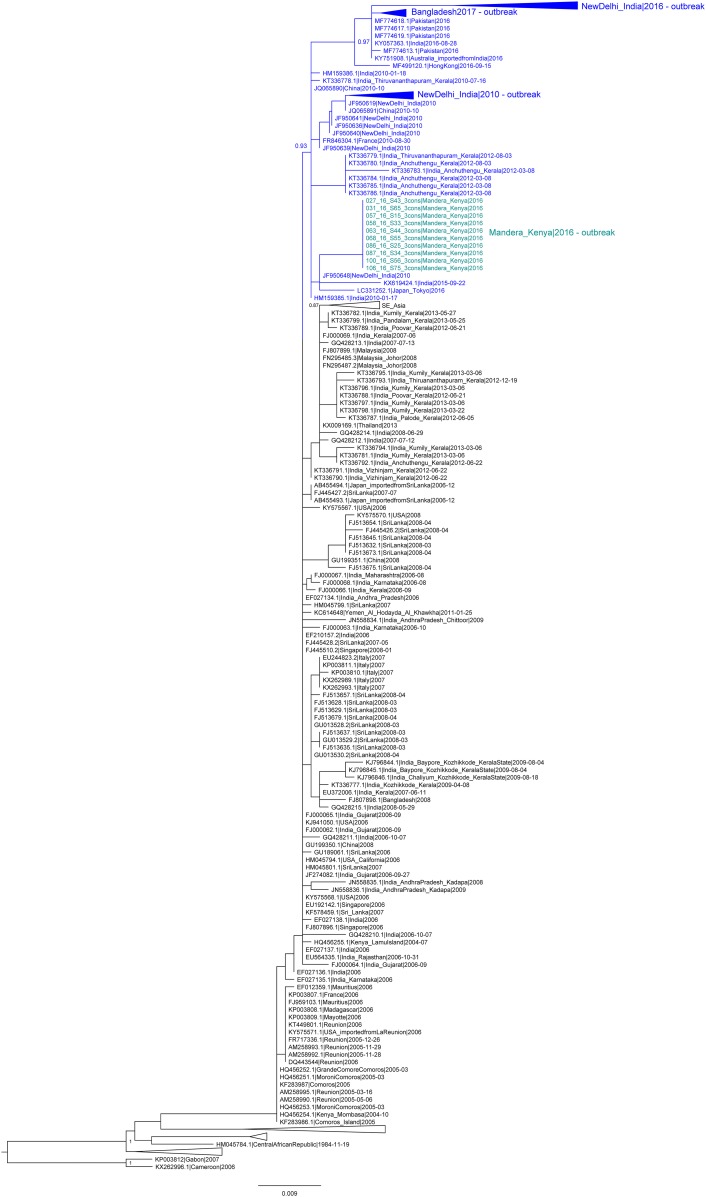
Partial E1 gene maximum likelihood tree of the chikungunya virus East, Central, and South African lineage. Taxa in blue (light blue for Kenya) represent genomes with the *Aedes aegypti* fitness-associated E1:K211E mutation. This figure appears in color at www.ajtmh.org.

Maximum likelihood trees of the partial E1 region, including additional sequences from the CHIKV outbreaks in 2010 and 2016 from New Delhi, India, and from 2017 in Bangladesh, showed that genomes from these outbreaks also belonged to the cluster with the *Ae. aegypti* fitness-associated mutations (Figure 2). Despite low phylogenetic signal due to the shorter E1 segment, the cluster was supported by a high confidence value, 0.93. These results indicated that, in addition to the outbreaks of Pakistan and Kenya, the more recent outbreaks in India and Bangladesh were also most probably caused by the variant carrying mutations with possible increased fitness to *Ae. aegypti.*

### Positive selection was acting on the E1:K211 but not the E2:264 position.

To investigate whether the E1:K211E and E2:V264A mutations appeared because of selective pressure and adaptation of the virus, we performed several tests for the presence of positive selection, on both the large CHIKV dataset used for the ML trees and on the smaller dataset used for BEAST analyses. Significant presence of positive selection in position E1:K211 was detected by FUBAR (probability > 0.99), FEL (*P* ≤ 0.05), and MEME (*P* < 0.05) in all tested datasets. Position E2:264 did not show any evidence of positive selection. No branch-specific selection was detected by aBSREL. Positions found to be under positive selection by all methods are listed in Table 2.

**Table 2:** Positively selected positions by method

	FUBAR	FEL	MEME
Maximum likelihood dataset	E1:211	nsP1:171, nsP3:117, E1:211	nsP1:4, nsP1:82, nsP1:157, nsP1:171, nsP1:301, nsP1:407, nsP2:349, nsP2:457, nsP2:604, nsP3:117, nsP3:303, nsP4:81, nsP4:467, nsP4:605, E2:57, E2:178, 6K:47, E1:146, E1:211, E1:382
BEAST dataset	E1:211	nsP1:171, nsP3:117, E1:211	nsP1:4, nsP1:101, nsP1:171, nsP2:349, nsP3:117, nsP4:81, nsP4:467, E2:57, 6K:47, E1:146, E1:211

### E1:K211E and E2:V264A mutations introduce charge and hydrophobicity changes to the CHIKV E protein.

**Figure 3.**
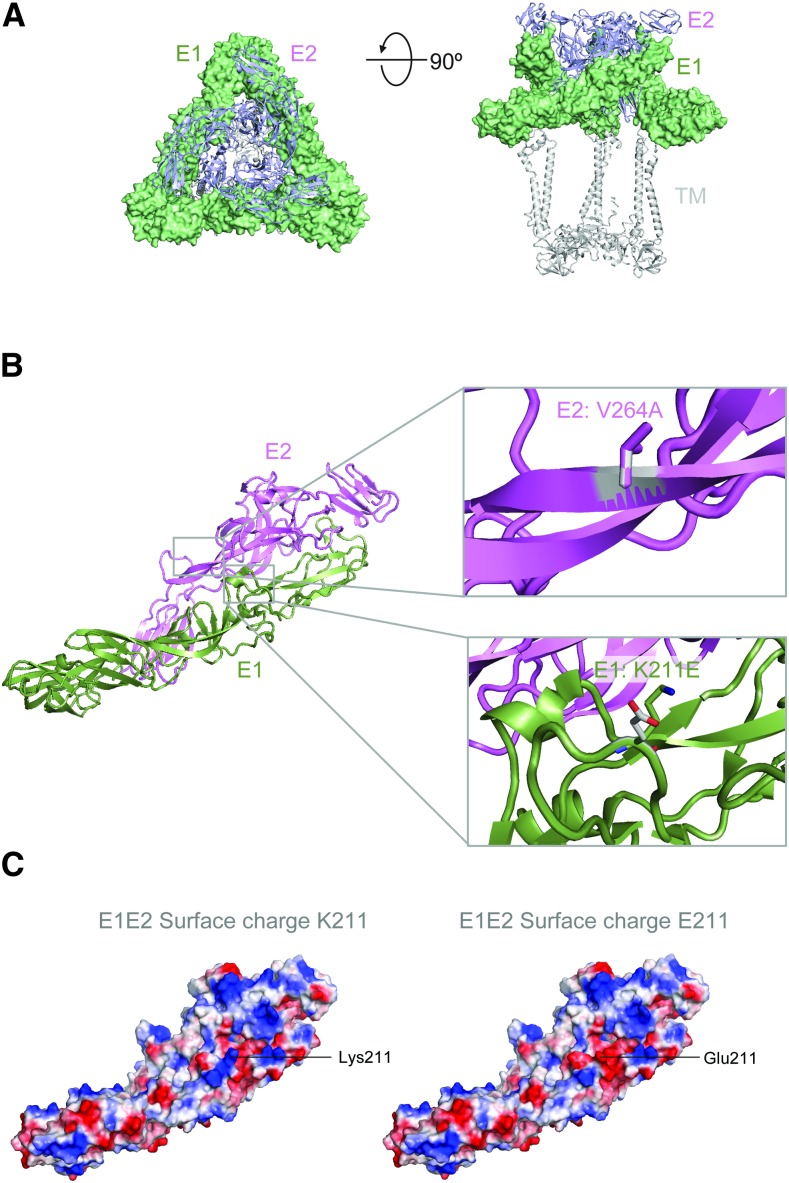
Structure analysis of chikungunya virus (CHIKV) mutations. (**A**) Structure of the CHIKV E1E2 viral surface glycoprotein trimer molecule^31^ (PDB ID: 3J2W) is shown in two orientations with E1 in surface representation (green) and E2 in ribbon representation (blue). (**B**) A single E1E2 heterodimer is shown in ribbon representation with close-up windows showing the V264A and K211E mutations in stick representation. (**C**) The E1E2 heterodimer is shown in surface representation with the surface colored by charge (blue: positive, red: negative, and white: neutral). The Lys211 (left) and mutant Glu211 (right) variants are modeled. This figure appears in color at www.ajtmh.org.

To investigate the potential impact of the E1:K211E and E2:V264A mutations on the structure of the CHIKV envelope protein, we modeled these mutations on a 3D structure of the E1E2 viral surface glycoprotein trimer molecule (Figure 3A).^31^ Our results show that both E1:211 and E2:264 residues are located centrally within the E1E2 heterodimer (Figure 3B). Our results also indicate that the K211E mutation causes a local change in the surface charge of the molecule (Figure 3C) from positive to negative. The V264A mutation reduces the hydrophobicity of this surface-exposed region.^34,35^

## DISCUSSION

Recent reemergence and global spread of CHIKV, coupled with its high morbidity and economic burden, has made it one of the more medically important arboviral diseases with major public health implications. Chikungunya virus originated in Africa, and the first known outbreak was recorded in today’s Tanzania in 1952–1953. Subsequently, sporadic outbreaks in Africa and larger epidemics in Asia were observed until the 1980s, followed by a period of decreased activity. The virus reemerged in the early 2000s in Africa, resulting in extensive and rapid spread throughout the world. In this study, we analyzed 10 CHIKV genomes sampled from the 2016 outbreak in Mandera, Kenya, and compared them with the genomes from the Kenya outbreak in 2004. We estimated the time of the 2016 outbreak emergence, as well as traced the emergence and movement of the previously reported *Ae. aegypti* fitness mutations that characterized these genomes. Variants with increased vector fitness may have the potential for more efficient transmission, resulting in large outbreaks, and may outcompete wild-type viral variants in the regions with abundant vector populations.

Our results confirm previous findings that reemerging CHIKV reached Kenya in mid-2002.^3,36^ Here, the virus splits into two variants. One variant caused disease in Mombasa in 2004 and spread further to Comoros, Madagascar, and La Réunion, where it obtained the *Ae. albopictus*–adaptive mutation E1:226V. The other variant, still carrying the wild-type E1:226A, spread north, causing the Lamu Island outbreak. Although the Lamu Island outbreak was observed first and was the largest in Kenya in 2004, present results suggest it did not seed the virus in Mombasa. Rather, the two shared a common ancestor that split into two different directions. Given that only one genome from each region is available, more data would be needed to completely resolve virus movement within Kenya during this period of time. Exact estimations of intercontinental routes of geographic spread are also limited by sampling skew; however, the wild-type variant from Lamu Island was next observed causing outbreaks in 2005–2006 in India, mainly on the west and south of the country (Rajasthan, Gujarat, Maharashtra, Andhra Pradesh, and Karnataka).^37–39^ Shortly thereafter, in 2007, a variant carrying the *Ae. albopictus*–adaptive E1:226V mutation was recorded in the province of Kerala.^40^ Both variants were found circulating in the following years in the country, between 2007 and 2013.^41,42^

In India, both *Ae. albopictus* and *Ae. aegypti* are prevalent vectors, and the presence of the recently described *Ae. aegypti* fitness-enhancing mutation E1:K211E was first observed in the Kerala–Puducherry CHIKV outbreak of 2006.^12^ Shortly thereafter, E1:K211E was accompanied by the second *Ae. aegypti* fitness-associated mutation, E2:V264A. This novel dual mutation variant was first observed in Kerala in 2009; however, the viruses from the Kerala–Puducherry CHIKV outbreak of 2006 were not sequenced in the E2 region.^13^ These results support our estimate that the *Ae. aegypti* variant carrying the E1:K211E and E2:V264A mutations probably arose in India sometime between 2005 and 2008. We show that this variant evolved directly from the E1:226A wild-type virus, and not from the E1:A226V virus with increased transmission efficiency by *Ae. albopictus.* In fact, the E1:K211E and E2:V264A mutations have not yet been detected in the naturally occurring *Ae. albopictus*–adapted virus. However, it is worth investigating whether the whole reemerging ECSA genotype virus has other amino acid residues that make it generally more fit for spread by *Ae. albopictus*.^43^ In that case, the two reported *Ae. aegypti* fitness mutations might represent compensatory adaptation of ECSA CHIKV, selected by reintroduction of an *Ae. albopictus*–adapted virus into regions infested with *Ae. aegypti*.

Our analyses suggest that the first indication of the E1:K211E and E2:V264A variant’s association with major outbreaks comes from India, where it caused an outbreak in New Delhi in 2010.^44^ Following this, CHIKV continued to circulate in discrete regions throughout India, and in 2016, the dual mutation variant appeared in another large outbreak in New Delhi.^45^ Simultaneously, it was also driving the outbreaks in Pakistan and Kenya.^46^ The Kenyan outbreak was estimated to have originated from a virus imported by 2015 and was most closely related to genomes from India. It is important to note that the implications of spread from India to Kenya are limited by sampling gaps, and India might not have been the direct source of the Kenyan outbreak. Indeed, the connection between the Somalian and Kenyan outbreaks indicates that the virus was most probably introduced from Somalia and, thus, that this variant is most likely spreading in African countries other than Kenya. Further sampling and sequencing in this region will reveal whether this is the case, or if the strong social, cultural, and human movement ties between Kenya and India aided in the dispersion of the virus between the two continents. Importantly, however, our results indicate that the variant carrying the two mutations with possible increased fitness to *Ae. aegypti* is no longer confined to India, but is spreading and associating with large outbreaks in other countries and other continents of the world. Interestingly, all recent CHIKV ECSA lineage genomes from the *Ae. aegypti* prevalent regions of the Indian Ocean sub-lineage contain the two *Ae. aegypti* fitness mutations. More sampling and surveillance are needed to determine whether the emergence of this new variant can thus lead to the replacement of the endemic wild-type virus.

After the 2016 Kenyan outbreak resolved, a new CHIKV outbreak started in the coastal city of Mombasa in 2017–2018, with a large proportion of cases (70%) reporting severe joint pain and high fever.^47^ No publicly available genomes exist from this event as of yet, but given the proximity of the *Ae. aegypti*–associated dual mutation virus outbreak of 2016, and given this variant’s association with large outbreaks, it is very possible that it is also responsible for this recent occurrence in Mombasa. Furthermore, other simultaneous CHIKV outbreaks have been reported in proximate regions to both Kenya and India, the 2016 outbreak in Mozambique and the 2017 outbreak in Dhaka, Bangladesh.^48,49^ The virus from the Dhaka outbreak contained the K211E mutation, and despite the lack of E2 sequencing, our analyses of the E1 region placed it within the *Ae. aegypti* dual mutation cluster. Thus, these results suggested that this outbreak was also caused by the variant with mutations previously associated with increased CHIKV fitness for *Ae. aegypti*.^49^ Sequencing and analyzing complete viral genomes from these and other countries will aid in the tracking of this variant’s spread, and it will provide insight into its possible replacement of the wild type in the *Ae. aegypti*–prevalent regions of the world.

The epidemic potential of a vector-borne agent depends on several interconnected intrinsic and extrinsic factors, such as viral and vector genetics, vector and host competence, vector abundance, temperature, and rainfall.^50^ Viral genetics, and the appearance of adaptive vector mutations, has previously been shown to play a role for the vector competence and spread of CHIKV by *Ae. albopictus*.^4^ The genetic impact of the recently described E1:K211E and E2:V264A mutations for the spread of CHIKV by *Ae. aegypti* is still not very well known. Our structural modeling suggests that these two mutations introduce changes in charge and hydrophobicity of the CHIKV envelope surface proteins. Previous studies in chikungunya and other alphaviruses indicate that changes in the E1/E2 proteins affect pH sensitivity and can have dramatic effects on structure and virus production levels.^51–53^ This phenomenon is seen with surface viral glycoproteins from other viruses as well, including HIV-1 and respiratory syncytial virus, where variants with reduced conformational flexibility have improved expression.^54,55^ Vector competence has been shown to vary greatly between different populations of *Aedes* mosquitos, implying importance of vector genetics, in addition to changes in important viral surface protein residues, in driving the spread of CHIKV.^56,57^ Thus, the possible impact of E1/E2 structural changes by the E1:K211E and E2:V264A mutations on virus infectivity, dissemination, and transmission by *Ae. aegypti* in different parts of the world could differ, and should be examined further. However, the presence of positive selection on E1:K211E, coupled with the increased spread of E1:K211E and E2:V264A CHIKV variant throughout the world, highlights the importance and the urgency of such studies. The possible level of increased susceptibility of these vectors to CHIKV infection may provide information for implementation of additional or alternate vector-control strategies.

In conclusion, we show that the CHIKV variant, carrying the E1:K211E and E2:A264V mutations previously associated with increased fitness to *Ae. aegypti*, is capable of rapid spread in the regions of high *Ae. aegypti* presence. It has been associated with several recent large CHIKV outbreaks in Africa and Asia, showing capacity for global spread. Our study highlights the importance of further sampling, genomic surveillance, and vector competence studies to promptly assess the epidemic potential of this CHIKV variant.

## Supplementary Files

Supplemental appendix
