# Supplementary material for: Global Outbreaks and Origins of a Chikungunya Virus Variant Carrying Mutations Which May Increase Fitness for Aedes aegypti: Revelations from the 2016 Mandera, Kenya Outbreak
Source: Am J Trop Med Hyg. 2019 Mar 11;100(5):1249–57. doi: 10.4269/ajtmh.18-0980 (PMC6493958; doi:10.4269/ajtmh.18-0980)
Supplement: Supplementary file 1 [file tpmd180980.SD1.pdf]

Supplemental Materials and Methods

Samples
From May 2016, following reports of widespread incidence of febrile illness with severe joint pains in Mandera (a city at the border with Somalia) and its environs, samples were collected from suspected cases of all ages and sex, using standard phlebotomy practices by the Kenyan Ministry of Health (KMoH). Venous blood was collected in vacutainer tubes with no anticoagulant at health facilities and allowed to set then centrifuged to obtain serum for laboratory testing. Samples were transported via courier in cold chain to the laboratories at KEMRI where they were stored at -80^0 centigrade for testing. Specific RT-PCR primers were used to detect Chikungunya acute infection and subsequently, amplicons from a subset of Chikungunya RT-PCR positive samples were sequenced to confirm the RT-PCR results. The following RT-PCR testing procedures were used. Cell culture inoculations were performed on the samples to obtain isolates for in-depth studies.

Cell Culture Propagation
Vero cells were grown in 25-cm^2 cell culture flasks to 80% confluency in MEM containing 10% FBS, 2% glutamine, 100 U/mL penicillin, 100 µg/mL streptomycin, and 1 µL/mL amphotericin B. The cells were then rinsed with sterile phosphate-buffered saline (PBS), and 0.2 mL of clarified tick homogenate was added followed by incubation at 37°C for 45 min to allow virus adsorption. After incubation, MEM supplemented with 2% FBS, 2% glutamine, 100 U/mL penicillin, 100 µg/mL streptomycin, and 1 µL/mL amphotericin B was added into the flasks and the cells allowed incubating at 37°C for 14 days while observing cytopathic effects (CPE) on a daily basis. The supernatants of virus-infected Vero cell cultures exhibiting CPE of approximately 70% were harvested from the flasks for virus identification.

Nucleic Acid (RNA) Extraction
The QIAamp Viral RNA Minikit (QIAGEN, Hilden Germany) was used to extract viral RNA according to the manufacturer’s protocol. A final volume of 60 µL of RNA was obtained and used as a template for complementary DNA (cDNA) synthesis.

cDNA Synthesis from Viral RNA
In a 200 µl PCR tube, 10 µL of the extracted sample RNA was mixed with 2 µL of 50 ng/µL random hexamer primer and 1 µL of 10 mM deoxynucleotide solution (dNTPs), and incubated in a thermocycler for 5 minutes at 65°C and immediately chilled for 1 minute at 4°C. The following components were then added to the PCR tube: 4 µL of 5X First Strand Buffer (Invitrogen), 1 µL of 0.1 M DTT, 1 µL of RNAse OUT™ (40 U/µL) and 1 µL of Superscript III Reverse transcriptase (200 U/µl). The mixture was then incubated in a thermocycler for 5 minutes at 25°C, 50 minutes at 50°C and 15 minutes at 70°C. A total of 20 µL cDNA was obtained.

PCR Amplification
The PCR amplification of targeted viral sequences in the cDNA was performed in a 25-µL reaction containing: 12.5 µl of Amplitaq Gold 360 PCR master mix (Applied Biosystems USA), 50 picomoles each of forward and reverse primer, 2 µl of the cDNA and 9.5 µl of DEPC-treated water to top up to 25 µl. Samples were first tested using alphavirus family primers (VIR2052F 5'-TGG CGC TAT GAT GAA ATC TGG AAT GTT-3' and VIR2052R 5'-TAC GAT GTT GTC GTC GCC GAT GAA-3') [22] and flavivirus family primers (FU1 5'- TAC AAC ATG ATG

GGA AAG AGA GAG AA-3' and CFD2 5'- GTG TCC CAG CCG GCG GTG TCA TCA GC-3') [23]. Samples testing positive with alphavirus family primers were further tested with Chikungunya specific primers (7028 forward (5'-TGCGCGGCCTTCATCGGCGACTAC-3' and 8288 reverse (5'-CCAGGTCACCACCGAGAGGG-3')). In all the PCR reactions, appropriate positive control cDNA and a negative control were included. Electrophoresis of the amplified DNA products was done on a 1- 2% agarose gel in 1% Tris-borate EDTA buffer stained with ethidium bromide. PCR product bands were visualized using a UV transilluminator and recorded using a gel photo imaging system.

Sanger and confirmation

Amplicons were purified using Wizard SV Gel and PCR Clean-Up System kit (Promega Madison, WI, USA) and sequenced using the ABI-PRISM 3130 Genetic Analyzer (Applied Biosystems, Foster City, CA). Partial structural genes were sequenced using primers CHIK 7028F: 5' TGCGCGGCCTTCATCGGCGACTAC 3' (Position: 7043 to 7066 based on Lamu33 strain, HQ456255.1) and CHIK 8288r: 5' CCAGGTCACCACCGAGAGGG 3’ (Position: 8301 to 8282 based on Lamu33 strain, HQ456255.1), targeting a region of ~1260 bp. Raw chromatogram files for both forward and reverse sequences were edited for bad calls in Chromas version 2.4.4 Basic Local Alignment Search Tool was used to search the Gene Bank and confirm identity of the virus isolates. Sequence alignment was accomplished using the Muscle algorithm. The phylogenetic tree was inferred using the Maximum Likelihood method based on the General Time Reversible Model (10000 bootstraps) in Molecular Evolutionary Genetics Analysis (MEGA) version 7.0.
